# Localization, quantification and interaction with host factors of endogenous HTLV-1 HBZ protein in infected cells and ATL

**DOI:** 10.1186/s12977-015-0186-0

**Published:** 2015-07-04

**Authors:** Goutham U Raval, Carlo Bidoia, Greta Forlani, Giovanna Tosi, Antoine Gessain, Roberto S Accolla

**Affiliations:** Department of Surgical and Morphological Sciences, School of Medicine, University of Insubria, Via Ottorino Rossi n.9, 21100 Varese, Italy; Département de Virologie, Institut Pasteur, 75015 Paris, France

**Keywords:** HTLV-1, HBZ, Adult T cell leukemia, Monoclonal antibodies

## Abstract

**Background:**

Human T cell lymphotropic virus type 1 (HTLV-1) is the etiological agent of a severe form of neoplasia designated Adult T cell Leukaemia (ATL). It is widely accepted that the viral transactivator Tax-1 is the major viral product involved in the onset, but not in the maintenance, of neoplastic phenotype, as only 30–40% of ATL cells express Tax-1. It has been recently demonstrated that HBZ (HTLV-1 bZIP factor), a protein encoded by the minus strand of HTLV-1 genome, constantly expressed in infected cells and in ATL tumor cells, is also involved in the pathogenesis of leukaemia. The full role played by HBZ in oncogenesis is not clarified in detail also because of the limited availability of tools to assess quantitative expression, subcellular location and interaction of HBZ with host factors in ATL.

**Results:**

By the use of the first reported monoclonal antibody against HBZ, 4D4-F3, generated in our laboratory it has been possible to carefully assess for the first time the above parameters in HTLV-1 chronically infected cells and, most importantly, in fresh leukemic cells from patients. Endogenous HBZ is expressed in speckle-like structures localized in the nucleus. The calculated number of endogenous HBZ molecules varies between 17.461 and 39.615 molecules per cell, 20- to 50-fold less than the amount expressed in HBZ transfected cells used by most investigators to assess the expression, function and subcellular localization of the viral protein. HBZ interacts in vivo with p300 and JunD and co-localizes only partially, and depending on the amount of expressed HBZ, not only with p300 and JunD but also with CBP and CREB2.

**Conclusions:**

The possibility to study endogenous HBZ in detail may significantly contribute to a better delineation of the role of HBZ during HTLV-1 infection and cellular transformation.

**Electronic supplementary material:**

The online version of this article (doi:10.1186/s12977-015-0186-0) contains supplementary material, which is available to authorized users.

## Background

HTLV-1 is an oncogenic retrovirus responsible of a severe form of cancer, the Adult T cell Leukemia (ATL), characterized by the malignant transformation of CD4+ T cells [1]. HTLV-1 also causes a severe neurological disorder designated HTLV-1-associated myelopathy/tropical spastic paraparesis (HAM/TSP) [2]. Ten million people are infected worldwide although this number is certainly underestimated [3]. Like other retroviruses HTLV-1 produces structural proteins, Gag, Pol and Env, encoded by the plus strand of the viral genome (reviewed in [4]). The HTLV-1 genome expression is mainly influenced by two regulatory proteins, Tax-1 and Rex, encoded by the 3′ region of viral genome between *env* and 3′ LTR [4]. The viral protein Tax-1 is important for the transcription of the provirus and its oncogenic potential [5]. The minus strand of the viral genome encodes a transcript [6] whose protein product is designated HTLV-1 bZIP factor (HBZ) [7]. Interestingly, while Tax-1 is expressed only in 40% of cells from ATL patients, HBZ transcripts are constantly found in all ATL cells [4, 8]. This probably reflects the fact that HBZ is also important for infectivity and persistence in vivo [9]. HBZ contains a bZIP domain in addition to an activation (N-terminus) and a central domain [7]. There are two different isoforms of this protein: a spliced form containing 206 amino acids (sp1) and an unspliced form with 209 amino acids (us) [10, 11]. The sp1 form is more abundant and is found in almost all ATL patients [8]. Spliced HBZ is more potent than unspliced HBZ in inhibiting transcription from viral 5′ LTR. Indeed, experiments using cells transfected with tagged HBZ have shown that HBZ interacts with CREB-2 via its bZIP domain resulting in strong inhibition of the CREB-2/Tax-1 interaction instrumental for the activation of HTLV-1 LTR [7]. In addition to interacting with CREB-2, similar experiments have shown that HBZ binds to different proteins of the JUN family via its bZIP domain [12]. The binding to JunB and cJun induces a sequestration of these factors in nuclear bodies or an accelerated degradation of them. As a result, HBZ reduces the cJun/JunB-mediated transcriptional activation of a series of genes. Conversely, the binding of HBZ to JunD does not inhibit the JunD-mediated transcriptional activation of target genes; indeed HBZ-JunD complex upregulates even the expression of HBZ encoding gene [13, 14]. Interestingly, in many cases HBZ exerts opposite effects with respect to Tax-1 on signaling pathways (reviewed in [15]). HBZ interacts with the KIX domain of p300/CBP to deregulate their interaction with cellular factors. This interaction strongly affects also the Tax-1-dependent, p300/CBP-mediated viral transactivation [16]. HBZ inhibits, while Tax-1 activates, the classical Nuclear Factor kappa B (NFkB) pathway by inducing PDLIM2 expression which brings about proteasomal degradation of RelA [17]. HBZ suppresses, while Tax-1 activates, Wnt pathway by interacting with the disheveled-associating protein with a high frequency of Leucine residues (DAPLE) [18]. HBZ inhibits production of Th1 cytokines (particularly IFN-γ) by interacting with NFAT and thus impairing cell-mediated immunity [19]. A number of effects suggest an important action of HBZ in supporting and/or maintaining the proliferation of HTLV-1 infected cells and by this the initiation and persistence of ATL. For example, the interaction of HBZ with JunD activates the telomerase by up-regulating the expression of hTERT [20]. HBZ interacts with ATF3 and reduces the interaction of ATF3 with p53, possibly interfering with p53 signaling leading to apoptosis and thus increasing the potential of ATL cells to proliferate [21]. HBZ interacts with Smad3 and C/EBPα in a ternary complex which suppresses C/EBPα signaling pathway, again favoring proliferation of ATL cells [22]. Moreover, the capacity of HBZ to participate in ternary complexes with Smad3 and its interacting factors, such as p300, may explain the up-regulation of TGFβ signaling pathway that is activated by Smad3-p300. Interestingly TGFβ may increase the expression of FoxP3, a marker of regulatory T cells (Tregs) which can be infected by HTLV-1 [23]; this may predispose to the onset of ATL since the CD4^+^Foxp3^+^ Treg cell phenotype has been found in ATL leukemic cells [24, 25] although this aspect is still controversial [26].

Most of the reported sub-cellular localizations, biochemical interactions with cellular components and functional aspects related to HBZ have been assessed on cells transfected with HBZ in which the protein is usually overexpressed. The lack of suitable reagents such as monoclonal antibodies against the native, endogenous HBZ has not permitted to corroborate and validate all the functions attributed to HBZ, particularly in naturally HTLV-1 infected cells and in tumor cells of ATL patients. Here we began to fill-up this gap by generating a monoclonal antibody that can specifically react with endogenous HBZ in HTLV-1 infected and in ATL tumor cells. This has allowed a more precise definition of the subcellular localization of HBZ, its interaction with cellular factors and an estimate of its amount in vivo both in HTLV-1 infected and in ATL tumor cells.

## Results

### 4D4-F3, the first reported monoclonal antibody specifically recognizing HBZ protein

Spleen cells of a mouse immunized with GST-tagged HBZ were fused with myeloma cells (P3U1) using PEG (polyethylene glycol). The resulting hybridomas were selected in a HAT medium and their supernatants screened by western blot against the immunizing antigen. Out of the 96 hybridomas screened, one designated 4D4 showed strong positivity. The specificity for HBZ was demonstrated by its reactivity for a differently tagged HBZ protein (myc-HBZ) (Additional file 1: Figure S1). The 4D4 hybridoma cells were cloned by limiting dilution and supernatants from single clones were screened by ELISA. Among various positive clones, we further selected the 4D4-F3 clone for its strong reactivity also in western blotting analysis (Additional file 1: Figure S1). 4D4-F3 monoclonal antibody (mAb) was isotyped as IgG1 with kappa light chain by ELISA.

**Figure 1:**
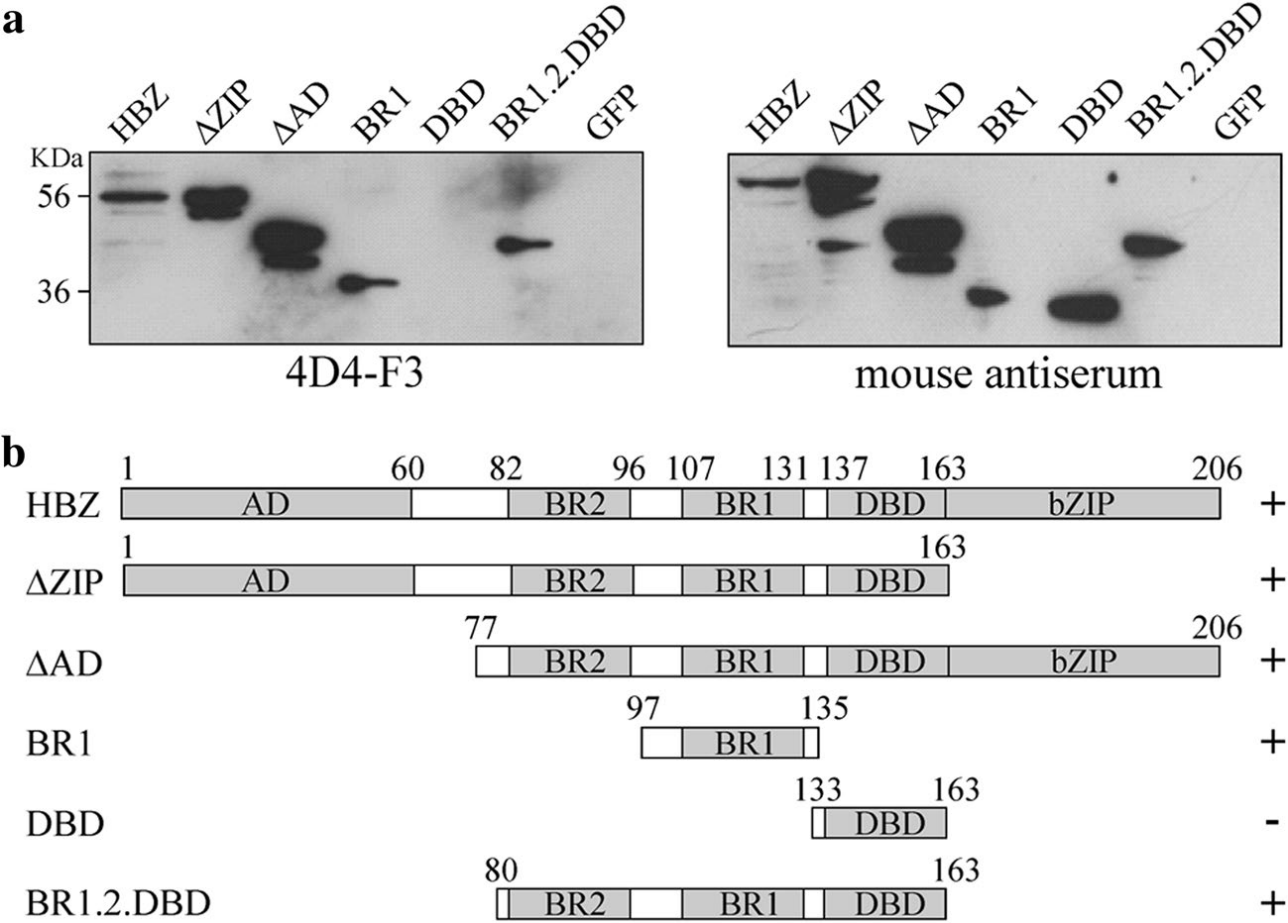
Epitope assignment of 4D4-F3 anti-HBZ mAb. **a** Western blot analysis of full length GFP-tagged HBZ (HBZ) and a series of GFP-tagged HBZ fragments listed in the top of the *left* and *right* *panels*. Cos cells were transfected with the various encoding GFP-HBZ cDNAs, with empty plasmid (mock) or with GFP cDNA (GFP). Twenty-four hours after transfection cell lysates were prepared and analyzed by western blotting using the 4D4-F3 anti HBZ mAb (*left panel*) or the anti-HBZ antiserum prepared from the same animal used for somatic cell fusion (*right panel*). **b** Schematic representation of the same results depicted in **a**. The various HBZ constructs are listed on the *left side* followed by *bars* representing size and regions of the specific fragment analyzed. *Numbers* indicate the specific aminoacid position in the various fragments. *Plus* (+) and *minus* (−) symbols indicate positive or negative reactivity, respectively, of the 4D4-F3 mAb. *AD* activation domain, *BR1* basic region 1, *BR2* basic region 2, *DBD* DNA binding domain, *bZIB* b zipper domain.

In order to delineate the HBZ epitope region recognized by 4D4-F3, an epitope mapping was carried out. For this purpose lysates of Cos cells transfected with either Green Fluorescent Protein (GFP)-tagged HBZ or a series of GFP-HBZ deletion mutants were analyzed by western blot. 4D4-F3 mAb recognized all fragments including the basic region 1 (BR1) fragment of HBZ, the isolated diagnostic BR1 fragment but not the isolated DBD fragment (Figure 1a, left panel). The presence of the DBD fragment in the corresponding lysate and its immunogenicity was confirmed by the reactivity of the serum of the mouse whose spleen cells were used for fusion (Figure 1a, right panel). Based on the above results the epitope recognized by 4D4-F3 mAb was mapped to the region between amino acids 97–133 (Figure 1b) including the BR1 region. Figure 1b summarizes the results of epitope mapping, listing all the different HBZ deletion mutants used and their size.

### Subcellular localization of HBZ as assessed by the 4D4-F3 monoclonal antibody

**Figure 2:**
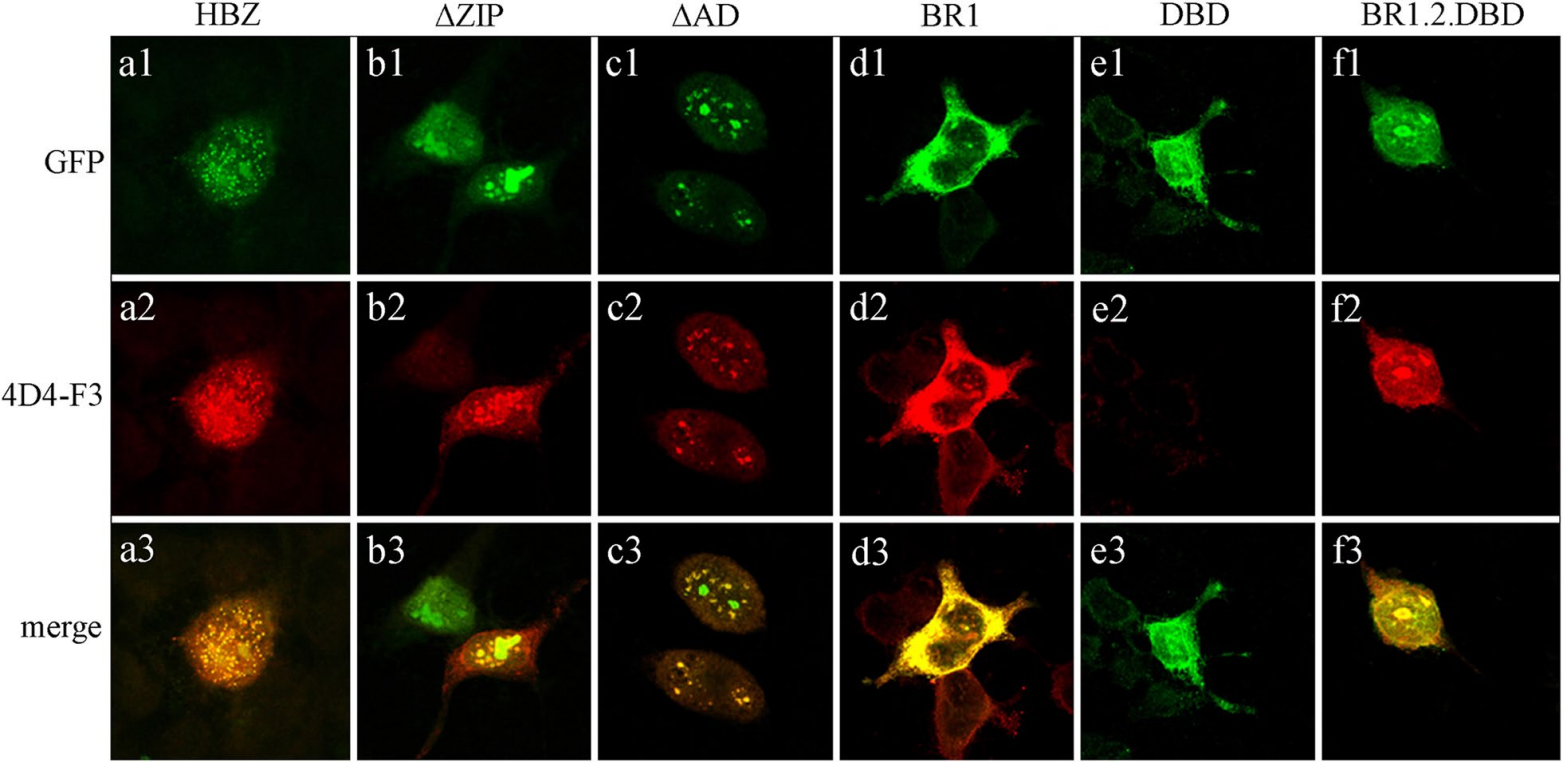
Subcellular localization of full length HBZ and HBZ fragments as assessed by the 4D4-F3 mAb. Full length GFP-tagged HBZ (HBZ) and a series of GFP-tagged HBZ fragments listed in the top of the figure, were transfected into 293T cells and their subcellular localization investigated by confocal microscopy either as GFP fluorescence (first series of *horizontal panels*, **a1**–**f1**) or as reactivity with the 4D4-F3 mAb followed by Alexa fluor 546-labeled goat anti-mouse antibody (second series of *horizontal panels*, **a2**–**f2**). Third series of *horizontal panels* represent the merge between the GFP and the Alexa fluor 546 signals (**a3**–**f3**).

**Figure 3:**
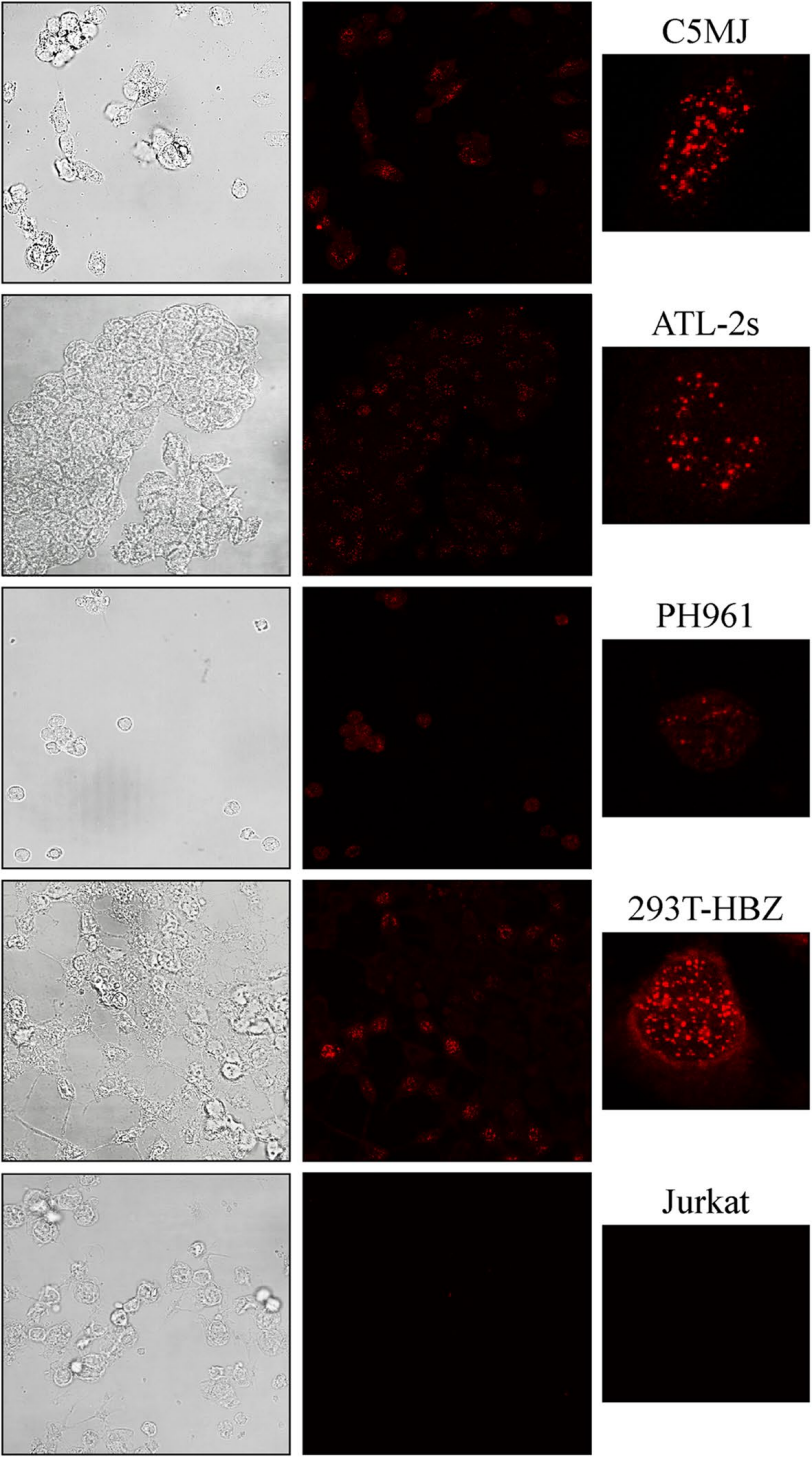
Subcellular localization of endogenous HBZ in HTLV-1 chronically infected and in ATL tumor cells. C5MJ and ATL-2s cell lines and PH961 patient PMBC were stained with the 4D4-F3 antibody, followed by Alexa fluor 546-labeled goat anti-mouse antibody and analyzed by confocal microscopy. 293T cells transiently transfected with myc-HBZ cDNA plasmid and the human T cell line Jurkat were used as positive and negative controls, respectively. *Right panels* show a representative single cell of each corresponding field. The percentage of HBZ-positive cells was calculated by counting at least 200 cells. Results were ATL-2s, 100%; C5MJ, 75%; PH961, 80%; 293T.HBZ, 55%.

4D4-F3 mAb was used to assess the localization and the distribution of HBZ, first in transfected cells and then in HTLV-1 infected and in ATL tumor cell lines. Previous studies using GFP-tagged HBZ or myc-tagged HBZ constructs transfected in COS or 293T cells, have documented a nuclear localization of HBZ in characteristic nuclear speckles [7, 12]. In our hands, GFP-HBZ transfected in 293T cells also showed a distribution in nuclear speckles (Figure 2a1). When the same cells were incubated with the 4D4-F3 mAb, a very intense staining was observed in the speckle-like nuclear structures virtually overlapping with the GFP-HBZ staining (Figure 2a2, a3, respectively). Interestingly, however, when GFP-HBZ deletion mutants were analyzed, a more refined and selective staining by the 4D4-F3 antibody was observed. This was particularly evident for the GFP-∆ZIP and GFP-∆AD nuclear fragments that often displayed strong green fluorescence staining in large nuclear aggregates. These structures were not observed with 4D4-F3 that instead detected HBZ in speckle-like structures (Figure 2, compare b2 and c2 with b1 and c1, respectively). This result strongly suggests that the GFP-related aggregates do not reflect a true HBZ nuclear distribution, possibly representing an artifact due to GFP. Virtual overlap in the staining between GFP and 4D4-F3 antibody was instead found for the predominantly cytoplasmic BR1 and the predominantly nuclear BR1-BR2-DBD HBZ fragments (Figure 3d1, d2 and f1, f2, respectively). Importantly, the 4D4-F3 antibody did not stain the GFP-DBD fragment (Figure 3e2) further confirming the results of the epitope mapping.

### Pattern of expression of endogenous HBZ protein in HTLV-1 infected and in ATL tumor cells

It was then investigated the crucial aspect of detection and cellular distribution of endogenously made HBZ in HTLV-1 infected cells and in ATL tumor cells. To this purpose, three cell types were used: the HTLV-1 chronically infected C5MJ cell line, the ATL-2s cell line derived from a patient with Adult T cell leukemia, and importantly, one fresh sample of peripheral blood mononuclear cells (PBMC) from a patient suffering of acute ATL. Preliminary analysis of the cell surface phenotype of C5MJ, ATL-2s and patient PH961 cells showed that C5MJ expresses CD4 and CD25 but not CD3, while ATL-2s did not express appreciable amounts of any of these markers. On the other hand, PBMC of the ATL patient PH961 expressed CD3 and CD25 in 100% of the cells and CD4 in 26% of the cells (Additional file 2: Figure S2). This rather diversified pattern of expression of T cell markers in HTLV-1 infected and in ATL cells has been previously observed by other investigators [27, 28]. Results of the confocal microscopy analysis are shown in Figure 3. In the two cell lines and in PBMC from ATL patient PH961, HBZ was detected in the characteristic speckle-like distribution in the nucleus, although the number of nuclear speckles was clearly lower as compared to that found in 293T cells transfected with untagged HBZ. No specific stain in the cytoplasm was detected. In addition, no signal was detected by the 4D4-F3 mAb in HBZ-negative Jurkat T cells, supporting the specificity of the monoclonal antibody. The speckle-like distribution of HBZ is reminiscent of a similar distribution of promyelocytic leukaemia protein (PML) nuclear bodies (PML-NB). PML is important in HTLV-1 infection because it may act as coactivator for the HTLV-1 Tax oncoprotein, although without direct binding [29]. Thus we assessed whether HBZ co-localizes with PML-NB. Results show that HBZ nuclear speckles and PML-NB do not overlap (Additional file 3: Figure S3), thus indicating that HBZ does not co-localize with PML-NB.

Careful analysis of confocal images allowed estimating the average number of HBZ-positive cells in PBMC of ATL patient and in the two cell lines, in comparison with the average HBZ-positive cells detected in HBZ-transfected 293T cells. ATL-2s cells were all positive for HBZ expression. Similarly, at least 80% of the ATL patient PBMC stained positive with the anti-HBZ mAb although with variable intensity. Seventy-five percent of the C5MJ cells stained positive for HBZ. In the reported experiment, 55% of HBZ-transfected 293T cells stained positive for HBZ.

### Quantification of endogenous HBZ protein in HTLV-1 infected and in ATL tumor cells

**Figure 4:**
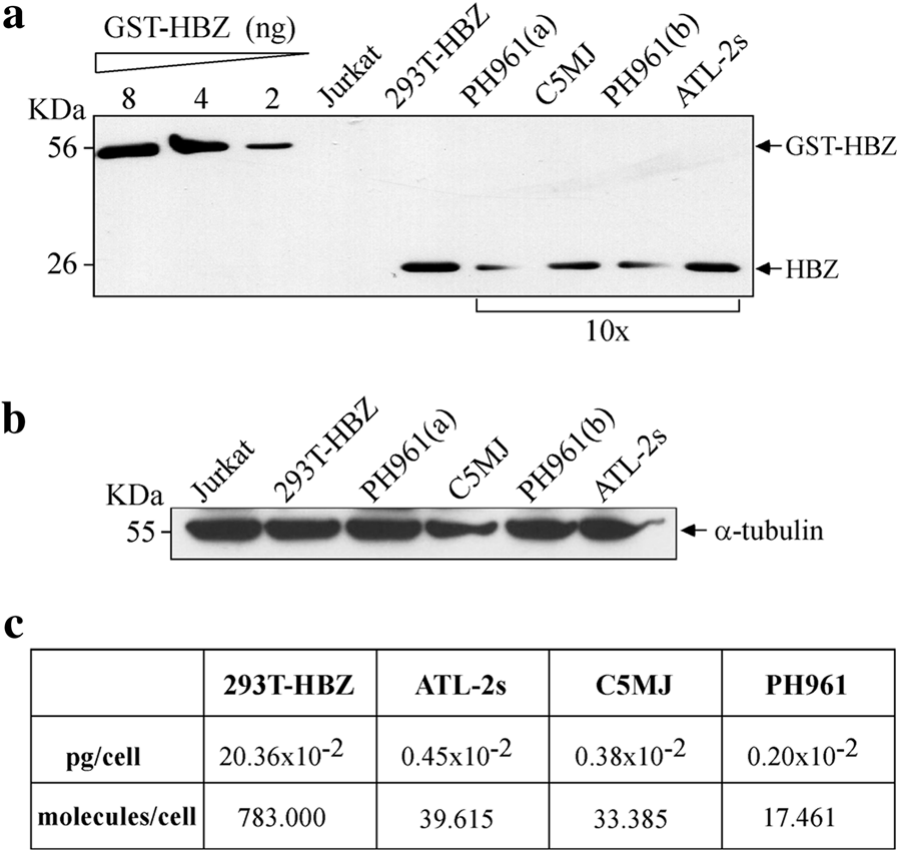
Quantification of endogenous HBZ expression in HTLV-1 chronically infected and in ATL tumor cells. **a** Three serial dilutions of purified GST-tagged HBZ protein (HBZ.GST) in nanograms (ng) were prepared in lysis buffer, run in SDS-PAGE gel, blotted in nitrocellulose filter, and filter reacted with the 4D4-F3 anti-HBZ mAb. Similarly, 40 μl of 1 ml cell lysates from C5MJ, ATL-2s, PH961 patient ATL tumor cells [PH961(a) and PH96(b)], two distinct cell lysates of equivalent number of cells, 293T cells transfected with HBZ-myc (293T.HBZ) and Jurkat cells, were run in the same gel and processed as above. Cell lysates were from the same cells analyzed by confocal microscopy and shown in Figure 1. It must be underlined that in order to obtain comparable western blots, C5MJ, ATL-2s and PH961 patient cell lysates were prepared from 10 times more cells as compared to 293T.HBZ cell lysates; this corresponded to 330.000 cells for the first three cell lysates vs 33.000 cells for 293T.HBZ cell lysate. Jurkat cell lysate was from 35.000 cells. **b** The expression of endogenous α-tubulin, used as internal control of sample loading. In this case, lysates from equal number of cells of the various samples were loaded in the gel. **c** The calculated amounts of HBZ in picograms/cell and in number of molecules/cell for the distinct cell sample are listed.

We further investigated whether the 4D4-F3 antibody could be used to estimate the amount of HBZ protein present in HTLV-1 infected and in ATL tumor cell lines. To this purpose, increasing amounts of purified GST-tagged HBZ protein were used to construct a standard curve by western blotting with the 4D4-F3 antibody; specific bands were then quantified by densitometry. As little as two ng of HBZ protein could be clearly detected by this approach (Figure 4a). Subsequently we performed western blot analysis of cell lysates from a fixed number of 293T cells transfected with the standardized dose of 3 μg of untagged HBZ DNA (Figure 4b) and plot the corresponding densitometry data on the curve constructed with the GST-tagged HBZ. This allowed defining a provisional value of the amount of HBZ present in the transfected cells on a per-cell basis. This value was calculated by taking into account the average percentage of HBZ transfected cells as shown in Figure 3, and it was found to be 20.36 × 10^−2^ pg/cell, corresponding to 783.000 molecules/cell. Similar experiments were performed with cell lysates of C5MJ and ATL-2s cell lines, as well as PH961 patient cells (Figure 4a). It must be stressed that in order to visualize by western blot specific HBZ bands in C5MJ, ATL-2s and in the PH961 patient cell lysates, comparable for intensity with HBZ-transfected 293T cells, it was necessary to process 10 times more cells. Assuming a molecular weight of 26 kDa for endogenous HBZ, after appropriate comparison by plotting densitometry values on the standard curve it was estimated that HBZ-positive C5MJ or ATL-2s cell lines contained an average of 0.65 × 10^−2^ pg/cell corresponding to 33.385 molecules/cell, and 1.03 × 10^−2^ pg/cell corresponding to 39.615 molecules/cell, respectively. For patients PH961 cells it was estimated an average of 0.45 × 10^−2^ pg/cell corresponding to 17.461 molecules/cell (Figure 4c). Therefore, with all the limitation of the analysis used, we could conclude that the amount of the endogenously made HBZ molecules in freshly isolated ATL cells, in ATL cell line and in a chronically infected cell line was comparatively much lower (20- to 50-fold lower) than the one observed in transiently transfected 293T cells.

### Endogenous HBZ interacts with several nuclear factors but co-localizes only partially with them

**Figure 5:**
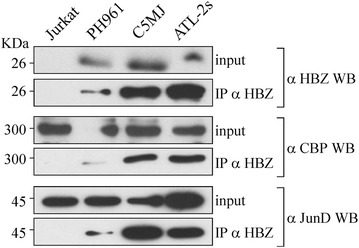
In vivo interaction of endogenous HBZ with intracellular molecules. In vivo interaction between endogenous HBZ and CBP or JunD was assessed by co-immunoprecipitation assay. Cell lysates from 50 million C5MJ, ATL-2s, PH961 ATL patient and Jurkat cells, prepared as described in the legend to Figure 2, were immunoprecipitated by using HBZ covalently linked to CNBr-activated Sepharose 4B beads (IP αHBZ). After elution from HBZ-Sepharose 4B beads, the eluted material was migrated on SDS-PAGE gels, blotted on nitrocellulose membranes and probed with antibodies specific for either anti-HBZ (4D4-F3 mAb, αHBZ WB), anti-CBP (αCBP WB) or anti-JunD (αJunD WB) (lower western blots in each series of *upper*, *middle* and *lower panels*, respectively). One twentieth of cell lysate from each sample was used to assess the presence of the specific protein (input) by western blot (upper western blot in each series of *upper*, *middle* and *lower panels*).

The 4D4-F3 anti-HBZ mAb was then used to assess the possible biochemical interactions and subcellular co-localizations of HBZ with cellular factors in the above-described ATL tumor cells and HTLV-1 chronically infected cells. Many nuclear factors have been reported to interact with HBZ. However, as pointed out earlier, most of these interactions were assessed in 293T or Cos cells transfected with tagged HBZ, in conditions of HBZ overexpression and in non- physiological cellular models of HBZ expression. We first checked the existence of in vivo interaction between HBZ and two crucial nuclear factors such as JunD and CBP that are involved in several steps of both HTLV-1 replication and regulation of the cellular gene expression. Cell extracts of C5MJ, ATL-2s and, importantly, of patient PH961 were first immunoprecipitated with the 4D4-F3 anti-HBZ mAb, and the resulting immunoprecipitate run in SDS-PAGE, blotted on filter and reacted with antibodies specific for either JunD or CBP. Results clearly indicate that endogenous HBZ interacts with both JunD and CBP (Figure 5), although to different extent depending on the cell analyzed and the amount of HBZ expressed. Indeed, both CBP and JunD interaction with HBZ were better detected in C5MJ and ATL-2s than in PH961 cells, and this correlated with the higher amount of HBZ expressed in the first two cells compared to the patient ATL tumor cells. As expected, over-expression of HBZ in 293T cells transfected with the viral gene was accompanied by a quantitatively higher interaction with both CBP and JunD, again suggesting that the limiting factor for biological interaction was the concentration of HBZ expressed by the cell.

**Figure 6:**
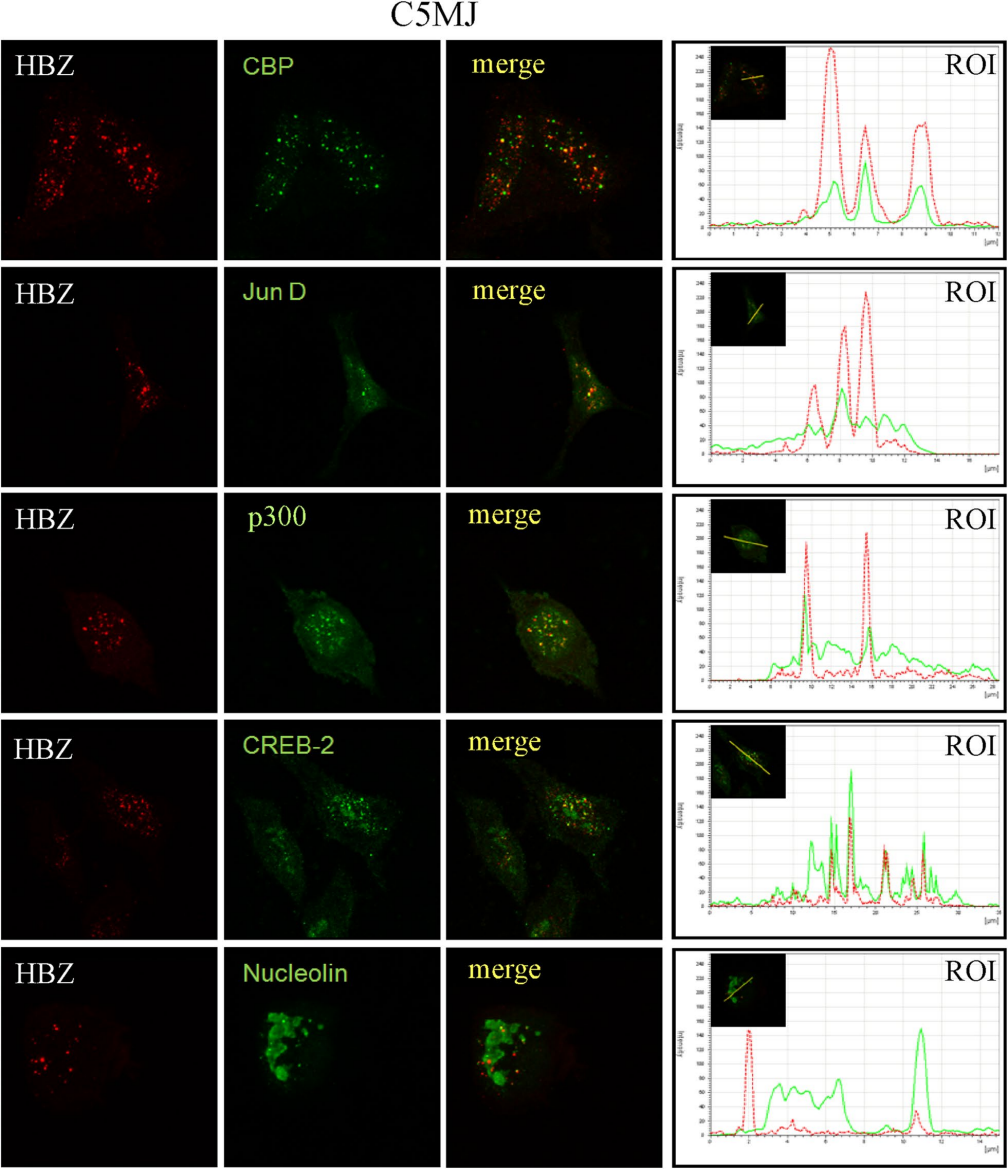
Co-localization of endogenous HBZ with intracellular factors in C5MJ cells. C5MJ cells were reacted in a pairwise combination with the 4D4-F3 anti HBZ mAb antibody, and either polyclonal rabbit anti-CBP, anti-JunD, anti-p300, anti-CREB2, or anti-nucleolin antibodies. Anti-HBZ mAb was revealed by Alexa fluor 546-labeled goat anti-mouse IgG (*red*), whereas the other rabbit antibodies were revealed by Alexa fluor 488-labeled goat anti-rabbit antibodies (*green*). The MERGE column panels represent the merge between the Alexa fluor 546 and the Alexa fluor 488 signals. A co-localization of HBZ with either one of the intracellular factors results in a *yellow color*. The reference of intensity (ROI) drawn along mid-nucleus level of the merge image (insert) is represented by *red* and *green peaks* in the histogram (*red* for HBZ and *green* for the intracellular factor).

**Figure 7:**
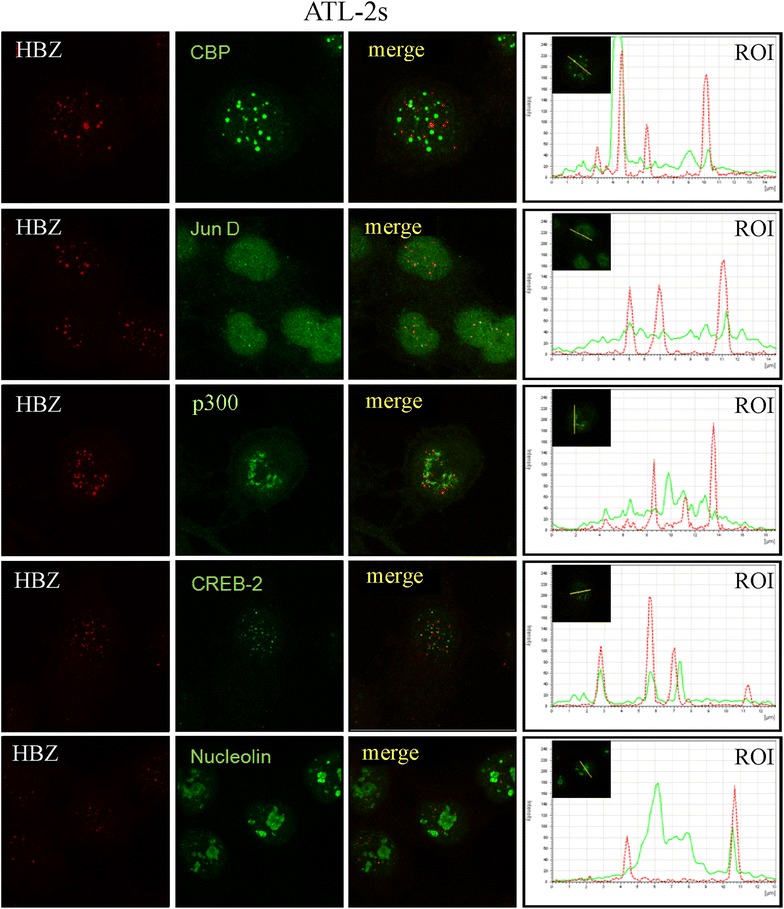
Co-localization of endogenous HBZ with intracellular factors in ATL-2s cells. ATL-2s cells were reacted in a pairwise combination with the 4D4-F3 anti HBZ mAb antibody, and either polyclonal rabbit anti-CBP, anti-JunD, anti-p300, anti-CREB2, or anti-nucleolin. For detection and other relevant informations see the legend to Figure 6.

**Figure 8:**
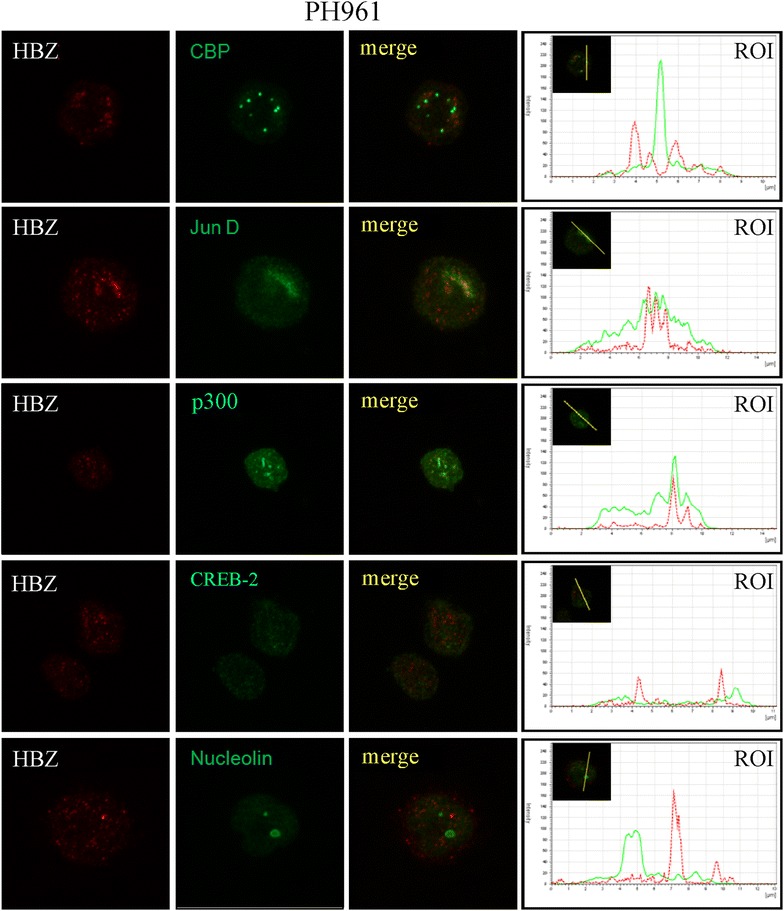
Co-localization of endogenous HBZ with intracellular factors in PH961 cells. PH961 patient cells were reacted in a pairwise combination with the 4D4-F3 anti HBZ mAb antibody, and either polyclonal rabbit anti-CBP, anti-JunD, anti-p300, anti-CREB2, or anti-nucleolin. For detection and other relevant information see the legend to Figure 6.

To further extend the study on the subcellular location of possible interaction between HBZ and cellular factors in HTLV-1 infected and ATL tumor cells, an extensive analysis by confocal microscopy was carried out. Moreover, confocal investigation was extended, besides CBP and JunD, also to p300 and CREB-2, as well as nucleolin a marker of nucleoli. Importantly, CREB-2 was one of the first identified cellular nuclear proteins interacting with HBZ via its bZIP domain and this interaction was shown to inhibit the CREB-2/Tax-1 interaction instrumental for the activation of HTLV-1 LTR and the subsequent viral replication. The results of such analysis are shown in Figure 6 for C5MJ, in Figure 7 for ATL-2s and in Figure 8 for patient PH961. In all figures, the first series of vertical panels depicts the distribution of HBZ protein (in red fluorescence), the second series of vertical panels the staining pattern of the various nuclear markers listed in each panel (in green fluorescence) and in the third series of vertical panels the merged fluorescence. The fourth series of vertical panels depicts the peak fluorescence distribution along a single line section (ROI) of the corresponding markers to better evidence specific co-localizations. Irrespective of the cell analyzed, a common feature was the relatively low co-localization of HBZ with each one of the various markers analyzed. Within this frame, co-localization between HBZ and CREB-2 in C5MJ (Figure 6) and ATL-2s (Figure 7) cell lines seemed to be more pronounced as compared to the one found in patients PH961 tumor cells (Figure 8). A second important feature was related to PH961 cells in which all markers showed a relatively lower expression compared to the two cell lines, probably reflecting the fact the immortalization and in vitro tumor growth induce a generalized overexpression of genes involved in the homeostasis of cell growth. Within the above-mentioned limits, HBZ co-localized with CBP and p300 better in C5MJ than in ATL-2s. JunD was diffusely expressed in ATL-2s with respect to both C5MJ and patient PH961 cells, rendering difficult the appreciation of HBZ-JunD co-localization in ATL-2s cells. Instead, a detectable co-localization between HBZ and JunD was observed in both C5MJ and patient PH961 cells. HBZ did not co-localize with nucleolin in any of the cells under study, indicating that the HTLV-1 viral protein is not a nucleolar protein.

## Discussion

HBZ is an important HTLV-1 viral oncogene that is strongly implicated in the neoplastic transformation leading to ATL [4]. In contrast to Tax-1 oncogene, required to initiate neoplastic transformation but not be to maintain the oncogenic process, HBZ is constantly found in ATL cells [15]. For lack of suitable reagents, however, this expression has been assessed mostly at the level of HBZ mRNA, and mRNA may not be the best suitable marker for HBZ protein expression [30]. In fact, previous reports have shown that HBZ protein is scarcely produced in the infected cells and is not, or only marginally, discriminated by HBZ specific CTLs [30, 31]. It is therefore extremely important to define its modality of expression, amount, localization and pattern of interaction with cellular factors during HTLV-1 infection and particularly in ATL cells. The above parameters can be defined in vivo only by using tools that can detect endogenous HBZ protein. Among these tools, specific monoclonal antibodies are certainly the most appropriate. Indeed, the use of the first described anti-HBZ mAb, 4D4-F3, has allowed us to better characterize the expression and the localization of HBZ molecules in chronically infected HTLV-1 positive cells as well as in cells from ATL patients, either as an established cell line (ATL-2s), or as fresh tumor cells from patient PMBC. Moreover, for the first time an estimation of the number of HBZ molecules expressed in HTLV-1 infected and ATL tumor cells could be obtained. 4D4-F3 mAb, raised against the full length spliced HBZ, was shown to react specifically with an epitope within the 97–135 aa region of the protein encompassing the BR1 region, strongly suggesting that this region is exposed on the outer surface of HBZ. The epitope recognized by the antibody encompasses a sequence present also in the unspliced form of HBZ. Thus, unless the small aminoacid difference between spliced and unspliced forms of HBZ at the N-terminus of the molecule [11] affects the conformation of the HBZ at distant sites, it would be expected that 4D4-F3 mAb recognizes both forms of the HTLV-1 viral protein.

Naturally expressed HBZ protein localized in the nucleus with a similar speckle-like distribution as the one observed in cells transfected with tagged HBZ protein. However several previously described aggregates of nuclear HBZ were shown to be artifacts of chimeric proteins, particularly in studies conducted with GFP-HBZ constructs. Moreover, HBZ-specific nuclear speckles did not overlap with similar speckle-like structures such as PML-NB involved in the co-activation of HTLV-1 Tax oncoprotein [29], indicating that HBZ does not co-localize with PML protein.

These findings emphasize the usefulness of our anti-HBZ mAb to better delineate the intracellular pattern of expression of HBZ. Confocal microscopy analysis of C5MJ, ATL-2s and particularly PBMC of the PH961 ATL patient indicated that the vast majority of the cells expressed HBZ. The percentage of positive cells varied from 75% in C5MJ, to 80% in PH961 ATL patient cells, and virtually 100% in ATL-2s cells. The results obtained in fresh PBMC of the PH961 ATL patient having a leukemic hyperlymphocytosis (400.000 cells/mm^3^) are important because they indicate that not only the vast majority of the PBMC are ATL tumor cells but also that virtually all of them express HBZ protein. PH961 patient ATL cells displayed a cell surface phenotype with 100% of the cells co-expressing CD3 and CD25 molecules, compatible with a T cell-activated phenotype, although these cells were only 26% positive for CD4 and negative for CD8 cell surface molecules. Thus most of the leukemic cells did not present the phenotypic characteristics of regulatory T cells (Tregs, CD4+/CD25+) or effector/memory T cells that are supposed to be the major targets of HTLV-1-mediated cellular transformation [24, 25, 32]. More refined studies are presently underway to further clarify the phenotype and the possible functional correlates of CD3+/CD25+/CD4− and CD3+/CD25+/CD4+ cells in PH961 patient. It must be stressed, however, that also the cell surface phenotype of the ATL-2s cell line was rather peculiar, as ATL-2s cells expressed very low amount, if any, of CD3 and CD4 molecules and only very limited amount of CD25 molecules (Additional file 2: Figure S2).

The availability of 4D4-F3 mAb has allowed for the first time to make an estimate of the number of HBZ molecules expressed on a per-cell basis in chronically infected C5MJ and in ATL tumor cells. In the three cells this number was far less (20- to 50-fold less) than the one expressed in HBZ-transfected cells such as Cos cells or 293T cells, used by most investigators to characterize the pattern of expression, the co-localization and the possible interaction of HBZ with cellular factors. In absolute number C5MJ, ATL-2s and patient PH961 cells contained 33.385, 39.615 and 17.461 molecules per cell, respectively, compared to an average of 783.000 molecules observed in HBZ-transfected 293T cells. These results may help to better understand and evaluate the quantitative expression of HBZ during the various phases of HTLV-1 infection leading to chronic infection and eventually to neoplastic transformation. Future investigation will be focused on these important aspects.

Many nuclear factors have been reported to interact with HBZ. However most of these interactions were assessed in 293T or COS cells transfected with tagged HBZ, thus in HBZ overexpression conditions and in non-physiological cellular models of HBZ expression. In this study we demonstrated that endogenous HBZ expressed in chronically infected and in ATL tumor cells interact in vivo with cellular transcription factors that are involved both in the control of HTLV-1 replication and in the host cell homeostasis. We could show that native HBZ can interact with CBP and JunD both in C5MJ and in the ATL tumor cells, although to different extent. In the two cell lines C5MJ and ATL-2s the interaction was consistently higher than the one observed in fresh ATL patient PH961 cells and partially correlated to the higher expression of HBZ protein in the former cell lines compared to the latter fresh tumor cells. These results conclusively demonstrate that native HBZ can indeed interact in vivo with CBP and JunD in situations of “physiological” expression of the viral protein both in chronically HTLV-1 infected cells and in ATL tumor cells. This study did not address the question of whether these interactions, as well as other described interactions such as the HBZ-CREB-2 and HBZ-p300, have a crucial functional role on the homeostasis of HTLV-1-infected cells and a causative implication in the cellular transformation leading to the ATL phenotype. Nevertheless, in C5MJ, ATL-2s and patient PH961 cells careful confocal microscopy analysis of co-localization of HBZ with either CBP, JunD, p300 or CREB-2 clearly demonstrated a partial, often only minor co-localization between the viral protein and the cellular nuclear factors. Thus, possible functional correlates of each single interaction of HBZ with the above nuclear factors in HTLV-1 infected and ATL cells should be framed within these findings.

## Conclusions

In conclusion, we believe that the availability of the 4D4-F3 anti-HBZ mAb may significantly contribute to a better delineation of the role and presence of HBZ during HTLV-1 infection and cellular transformation.

## Methods

### HBZ constructs

The HBZ plasmid pGEX2T-HBZ, used to prepare the recombinant GST-HBZ protein in its spliced form used for immunization (see below), was a gift of Dr. Lemasson, University East Carolina, USA [16]. Plasmid encoding myc-tagged HBZ, comprising also an histidine tag after the short myc sequence, was a gift of Dr. Matsuoka, Univ. of Kyoto, Japan. After expression, this recombinant protein has an apparent molecular weight of 36 kDa [8]. Full length and mutant fragments of GFP-tagged HBZ (pEGFPc2.HBZ plasmids) were provided by Dr. Mesnard, Montpellier, France [33]. Plasmid encoding untagged HBZ (pcDNA3.HBZ) was obtained by double digestion of the pEGFPc2.HBZ full length plasmid with XbaI and HindIII and ligation of the resulting HBZ sequence to corresponding restriction sites of the plasmid pcDNA3.1hygro (+).

### Recombinant HBZ protein production, purification and quantification

pGEX2T-HBZ plasmid was used to transform rosetta bacterial cells (rosetta-gami pLysS competent cells, Novagen, Merck Millipore, Germany). The cells were grown with ampicillin and chloramphenicol selection as suggested by the manufacturer. When the absorbance at 600 nm reached 1.0, 1 mM isopropyl beta-D-1-thiogalactopyranoside (IPTG) was added to induce the recombinant protein expression. After overnight incubation at 37°C, the culture was centrifuged to get the pellet. In order to lyse the bacterial cells we used a modified RIPA buffer (Tris–HCl 20 mM pH 8.5; NaCl 500 mM; EDTA 1 mM; Tween 20 0.2%; Protease inhibitor mix from Roche, Milan, Italy). The lysate was sonicated at 50% power, 30 s (0.9/0.1 pulse) for three times. The lysate was then centrifuged at 4,500 rpm for 30 min to get the supernatant and pellet. To purify the recombinant protein (HBZ-GST), agarose glutathione beads (Thermo Scientific, Rockford, USA) were mixed with the lysate obtained above and incubated overnight on a rotor wheel at 4°C. Next day, the beads were washed and the recombinant protein was eluted with elution buffer (Tris 50 mM, Nacl 150 mM pH 8, reduced glutathione 10 mM). All washes and eluants were stored at −20°C for further analysis. In order to quantify the fusion protein, a protein assay kit (BCA, Thermo Scientific) was used following manufacturer’s instructions.

### Cells

COS and 293T cell lines were cultured in DMEM medium, supplemented with 10% fetal calf serum (FCS) and glutamine. HTLV-1 chronically infected cell line C5MJ [34] was kindly obtained from Dr. Macchi, University of Rome Tor Vergata and maintained in RPMI-FCS 10%. The ATL tumor cell line ATL-2s was obtained from Dr. Matsuoka, Japan [35]. Peripheral blood monuclear cells (PBMC) PH961 were from a patient under the epidemiological control of the Pasteur Institute, Paris, France, with a typical acute ATL form characterized by a high level of circulating leukemic cells, very high hyperlymphocytosis with lymphocyte count of 400 000 lymphocytes/mm^3^. HTLV-1 infection was confirmed by Western blot on plasma sample and anti-HTLV-1 antibodies in patient’ plasma were titrated through indirect immunofluorescence using the HTLV-1-producing MT-2 cell line, as described previously [36]. Anti-HTLV-1 antibody titer of patient from patient PH961 was 1/10,240. Patient’s data were analyzed anonymously.

The immunophenotype of C5MJ, ATL-2s and patient PH961 PBMC was assessed by immunofluorescence and flow cytometry (BD FACSAria II™ apparatus) using the following monoclonal antibodies: mouse anti-human HLA class I (clone B9.12); mouse anti-human HLA class II DR (clone D1.12); mouse anti-human CD19 (clone HIB19, BD Pharmingen); mouse anti-human CD16 (clone 3G8, BD Pharmingen, Milan, Italy); the above antibodies were revealed by FITC-labelled rabbit anti-mouse IgG F(ab’)2 antiserum (Sigma, Milan, Italy); FITC mouse anti-human CD3 (clone (UCHT1 BD Pharmingen); FITC mouse anti-human CD4 (clone RPA-T4, BD Pharmingen); phycoerythrin (PE) mouse anti-human CD25 (clone M-A251, BD Pharmingen), and PE-Cy5 mouse anti-human CD8a (clone RPA-T8; eBioscience, Milan, Italy). Incubation and staining procedures were as previously described [37].

### Mice immunization and generation of anti-HBZ monoclonal antibody

All animal work has been conducted according to relevant national and international guidelines and was approved by the University of Insubria Internal Ethical Committee CESA (project 07-2013) and by the Italian Ministry of Health. Six-week-old female BALB/c mice were injected intraperitoneal on day 1 and day 20 with 10 µg of HBZ-GST protein in Complete Freund’s Adjuvant, total injected volume of 250 µl (125 µl PBS and 125 µl of Complete Freund’s Adjuvant). On days 43 and 68, HBZ-GST protein injections were repeated by using Incomplete Freund’s Adjuvant. Mouse serum was checked for anti-HBZ positivity on day 72 before sacrificing the animal. Total splenocytes were fused with 10 million P3U1 myeloma cells in presence of PEG as described [38]. The hybridomas were grown in selecting RPMI-HAT (Hypoxanthine Aminopterin Thymidine) medium. Initial screening of hybridoma supernatants was performed by an indirect enzyme-linked immunoadsorbent assay (protein detector ELISA kit, KPL). Briefly, 100 μl of purified GST-HBZ protein solution (1 μg/ml in PBS) was adsorbed onto the plate wells for 1 h at RT. Protein solution was removed and wells were saturated with 1× blocking solution for 1 h at RT. Hundred microliters of hybridoma supernatants were then added to the wells and incubated for 1 h at RT. After washing, peroxidase-labeled goat anti-mouse IgG was added and incubated for 1 h at RT. Finally, the substrate and stop solution were added and the plate was read at 405 nm in a spectrophotometer. The mouse serum from immunized animals was used as a positive control and the supernatant of P3U1 myeloma cells or normal mouse serum served as a negative control.

Hybridoma supernatants were also screened for anti-HBZ positivity by western blot. To this purpose cell lysates from Cos cells transfected with myc-HBZ (see below) was run on a 10% SDS-PAGE gel and transferred onto nitrocellulose membrane (Amersham high bond ECL, GE, Buckinghamshire, UK). The membrane was then quenched in 5% non-fat milk in 1× TBS containing 0.05% Tween-20 (MTTBS) for 1 h at room temperature. Hybridoma supernatant, diluted 1:1 with 5% MTTBS was then added and membrane incubated overnight at 4°C. After appropriate washes with MTTBS, HRP conjugated goat anti-mouse IgG was used as secondary antibody for 1 h at room temperature. The membranes were developed using the ECL system (Pierce, Rockford, USA) and exposed to X-ray film.

To determine the antibody isotype, we used Pierce rapid ELISA mouse mAb isotyping kit. Appropriately diluted hybridoma supernatant was added to the wells pre-coated with anti-mouse heavy or anti-mouse light chain capture antibody. Goat anti-mouse IgG + IgM + IgA HRP conjugate was then added to the wells and incubated for 1 h at room temperature. Following washes, TMB substrate and stop solution were added following manufacturer’ instructions and spectrofluorometric measure performed at 450 nm.

### Transfection procedures

Cos cells or 293T cells were transfected with full length HBZ constructs (GFP-HBZ, myc-HBZ, or untagged HBZ) or GFP-tagged HBZ fragments (∆ZIP, ∆AD, BR1, DBD, BR1-BR2-DBD) as previously described [39]. Briefly, 3 × 10^6^ COS or 293T cells were seeded in 35-mm-diameter plates and transfected with 3 μg of plasmid DNA constructs using Lipofectamine (Invitrogen, Milan, Italy). Cell extracts were prepared 24 h post-transfection and analyzed for the expression of recombinant proteins by SDS-PAGE and Western blotting with the anti-Myc 9E10 antibody (Santa Cruz Biotechnology) to detect myc-tagged HBZ. Horseradish peroxidase (HRP)-conjugated anti-mouse immunoglobulin secondary antibody (Amersham, Milan, Italy) was used. Blots were developed by chemiluminescence assay (Immune-Star HRP substrate; Bio-Rad, Milan, Italy).

### Confocal microscopy

Appropriate number of cells were plated onto a sterilized cover slip (pre-coated with D-polylysine 0.1 μg/ml, Sigma, Milan, Italy) placed in a six-well plate. After 24 h, transfection with 3 μg plasmid coding for HBZ-Myc or HBZ-GFP or different HBZ mutants was performed as above. The cells were then washed with PBS 1× three times and fixed with methanol (−20°C) for 4–6 min. Cells were washed three times with PBS, and incubated with a solution of 0.5% BSA in PBS for 1 h. The anti-HBZ mAb was then added as primary antibody and incubated overnight at 4°C. Alexa fluor 546 IgG1 goat anti-mouse was used as secondary antibody for detecting the monoclonal antibody. After three washes with PBS, the cover slip was mounted onto a glass slide using a drop of mounting medium and sealed. The images were visualized by a LeicaTCS SP5 confocal microscope (objective lenses: HCX PL APO, 63× original magnification, numerical aperture: 1.25) and imported into LAS AF software. A similar protocol was used for HTLV-1 infected cells C5MJ and ATL-2s. To assess co-localization of HBZ with host cellular factors C5MJ, ATL-2s and PH961 cells were reacted in a pairwise combination with the 4D4-F3 anti HBZ mAb antibody, and either polyclonal rabbit anti-PML, anti-CBP, anti-JunD, anti-p300, anti-CREB2, or anti-nucleolin, all from Santa Cruz Biotechnology, Ca, USA. Anti-HBZ mAb was revealed by Alexa fluor 546-labeled goat anti-mouse IgG (red), whereas the other rabbit antibodies were revealed by Alexa fluor 488-labeled goat anti-rabbit antibodies (green).

### Quantitative assessment of HBZ in chronically infected and in ATL tumor cell lines

Endogenous HBZ expression was quantitated by using the anti-HBZ monoclonal antibody 4D4-F3. To this purpose increasing amounts of purified GST-HBZ protein were migrated in SDS-PAGE, blotted with the 4D4-F3 antibody, and the intensity of resulting bands analyzed by densitometry to construct a standard curve. Cell lysates from endogenously HBZ-producing cells or HBZ-transfected cells were then assayed for the presence of HBZ by western blot; the intensity of the resulting bands was measured by densitometry as above and plotted on the densitometry standard curve obtained with purified GST-HBZ. Densitometry measurements and elaboration of data were carried out using the ImageJ software (Image Processing and Analysis in Java, http://imagej.nih.gov/ij/) The deduced concentration of HBZ in cells was obtained by taking into account the total number of cells used for preparing the cell lysate, the volume of cell lysate used and the percentage of positive cells visualized by confocal microscopy in parallel experiments. The obtained number in amount/cell was then converted in number of moles/cell taking into account the molecular weight of endogenous HBZ by the formula: [HBZ_gr_/HBZ_mw_ = HBZ_moles_]. Mole value (HBZ_moles_) was then transformed in number of molecules/cell by multiplying it for the Avogadro number following the formula: N_molecules_ = 6 × 10^23^ × HBZ_moles_.

### Co-immunoprecipitation

Lysates of ATL-2s, C5 MJ, PH961 cells, as well as lysate of HBZ-transfected 293T cells, or lysate from human T cell line Jurkat used as negative control, were prepared by using a non-denaturing lysis buffer (NP-40 1%, Tris-base 10 mM pH 7.4, Nacl 150 mM, EDTA 2 mM). Albumin-coupled Sepharose 4B beads (20 μl packed beads) were washed with the same lysis buffer and used to pre-clearing the lysates for 1 h at 4°C. For co-immunoprecipitation, the 4D4-F3 antibody was covalently linked to CNBr-activated Sepharose 4B beads following standard procedures. For this purpose 10 mg of purified 4D4-F3 mAb was covalently linked to 1 g dry weight Sepharose 4B as described [40]. Lysates were then incubated with 4D4-F3-coupled Sepharose 4B beads (10 μl packed beads/10 × 10^6^ cell equivalent) overnight at 4°C. 4D4-F3-coupled Sepharose 4B beads were washed six times with lysis buffer. Finally the beads were resuspended in 50 μl of loading buffer 2×, boiled and run on SDS-PAGE gel. After gel blotting on membranes, membranes were incubated with appropriate primary and secondary antibodies, exposed and developed as described above.

## Additional files

Additional file 1:
**Figure S1.** Specificity of the 4D4 parental and its cloned 4D4-F3 hybridoma for HBZ molecule. Cell lysates of Cos cells transfected with myc-tagged HBZ (HBZmyc) or non transfectd (mock) were migrated on SDS-PAGE gels, blotted on nitrocellulose membranes and probed with supernatant from the 4D4 parental hybridoma or its cloned product 4D4-F3, raised against a GST-tagged HBZ protein. As negative control, the supernatant of the P3U1 myeloma cells used for somatic cell fusion was used. Additional file 2:
**Figure S2.** Cell surface phenotype of C5MJ, ATL-2s and patient PH961 cells. Cell surface phenotype of C5MJ, ATL-2s cell lines and of peripheral blood mononuclear cells from ATL patient PH961 was assessed by immunofluorescence and flow cytometry. The various cell surface markers listed in the top right of each histogram were assessed by specific monoclonal antibodies either unlabeled (HLA class I and HLA class II DR) followed by incubation with FITC-labeled rabbit anti-mouse IgG, or directly labeled with fluorochromes (CD3, CD4, CD8, CD25, CD19, and CD16). Specific fluorescence is represented by the bold histogram; negative isotype control is represented by the thin histogram. Values are expressed in the abscissa as mean fluorescence in arbitrary units (au). Additional file 3
**Figure S3.** HBZ nuclear speckles do not co-localize with PML nuclear bodies (PML-NB). ATL-2s cells were reacted in a pairwise combination with the 4D4-F3 ant-HBZ mAb antibody and a polyclonal rabbit anti-PML antiserum. Anti-HBZ mAb was revealed by Alexa fluor 546 labeled goat anti-mouse IgG (red), whereas the rabbit antibodies were revealed by Alexa fluor 488-labeled goat anti-rabbit antibodies (green). Two representative cells are shown. The “merge” column panels represent the merge between the Alexa fluor 546 and the Alexa fluor 488 signals. A co-localization of HBZ with PML would result in a yellow color. Differential interference contrast (DIC) image of each cell analyzed is shown in the last column panels.

## Abbreviations

HTLV-1: human T cell lymphotropic virus-1
ATL: adult T cell leukaemia
HBZ: HTLV-1 b-ZIP factor
HAM/TSP: HTLV associated myelopathy/tropical spastic paraparesis
