# Supplementary figures and images for: Localization, quantification and interaction with host factors of endogenous HTLV-1 HBZ protein in infected cells and ATL
Source: Retrovirology. 2015 Jul 4;12:59. doi: 10.1186/s12977-015-0186-0 (PMC4491271; doi:10.1186/s12977-015-0186-0)

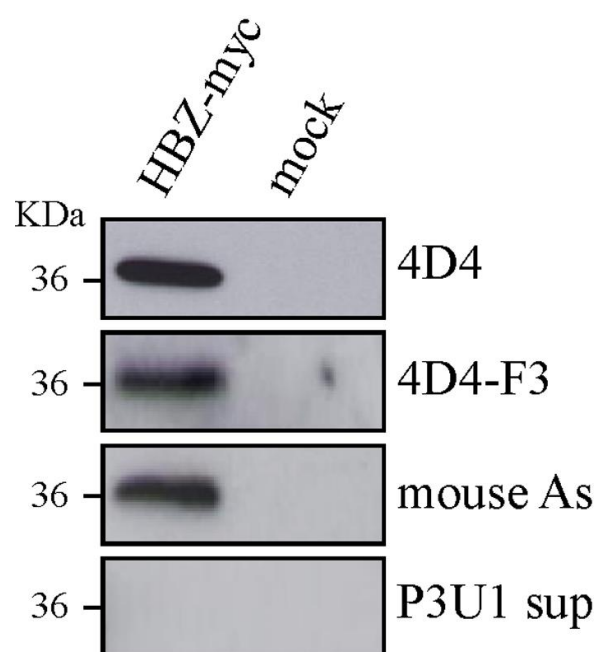

Supplement: Additional file 1: — Figure S1. Specificity of the 4D4 parental and its cloned 4D4-F3 hybridoma for HBZ molecule. Cell lysates of Cos cells transfected with myc-tagged HBZ (HBZmyc) or non transfectd (mock) were migrated on SDS-PAGE gels, blotted on nitrocellulose membranes and probed with supernatant from the 4D4 parental hybridoma or its cloned product 4D4-F3, raised against a GST-tagged HBZ protein. As negative control, the supernatant of the P3U1 myeloma cells used for somatic cell fusion was used. [file 12977_2015_186_MOESM1_ESM.pdf]

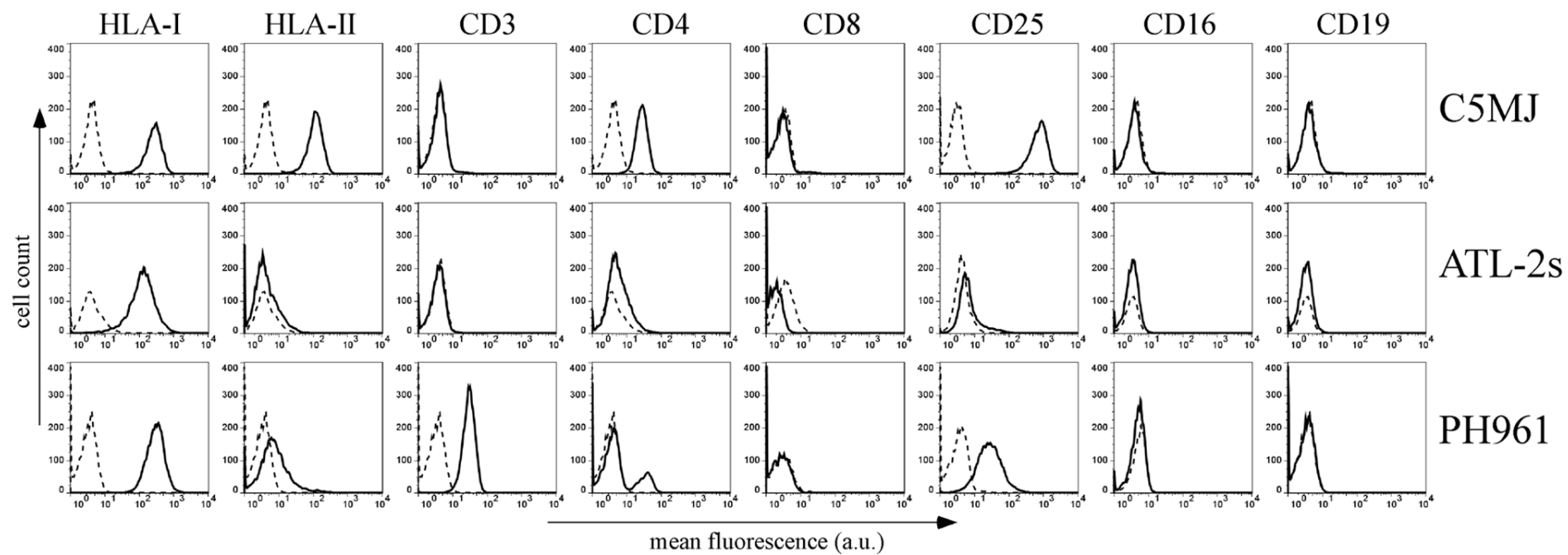

Supplement: Additional file 2: — Figure S2. Cell surface phenotype of C5MJ, ATL-2s and patient PH961 cells. Cell surface phenotype of C5MJ, ATL-2s cell lines and of peripheral blood mononuclear cells from ATL patient PH961 was assessed by immunofluorescence and flow cytometry. The various cell surface markers listed in the top right of each histogram were assessed by specific monoclonal antibodies either unlabeled (HLA class I and HLA class II DR) followed by incubation with FITC-labeled rabbit anti-mouse IgG, or directly labeled with fluorochromes (CD3, CD4, CD8, CD25, CD19, and CD16). Specific fluorescence is represented by the bold histogram; negative isotype control is represented by the thin histogram. Values are expressed in the abscissa as mean fluorescence in arbitrary units (au). [file 12977_2015_186_MOESM2_ESM.pdf]

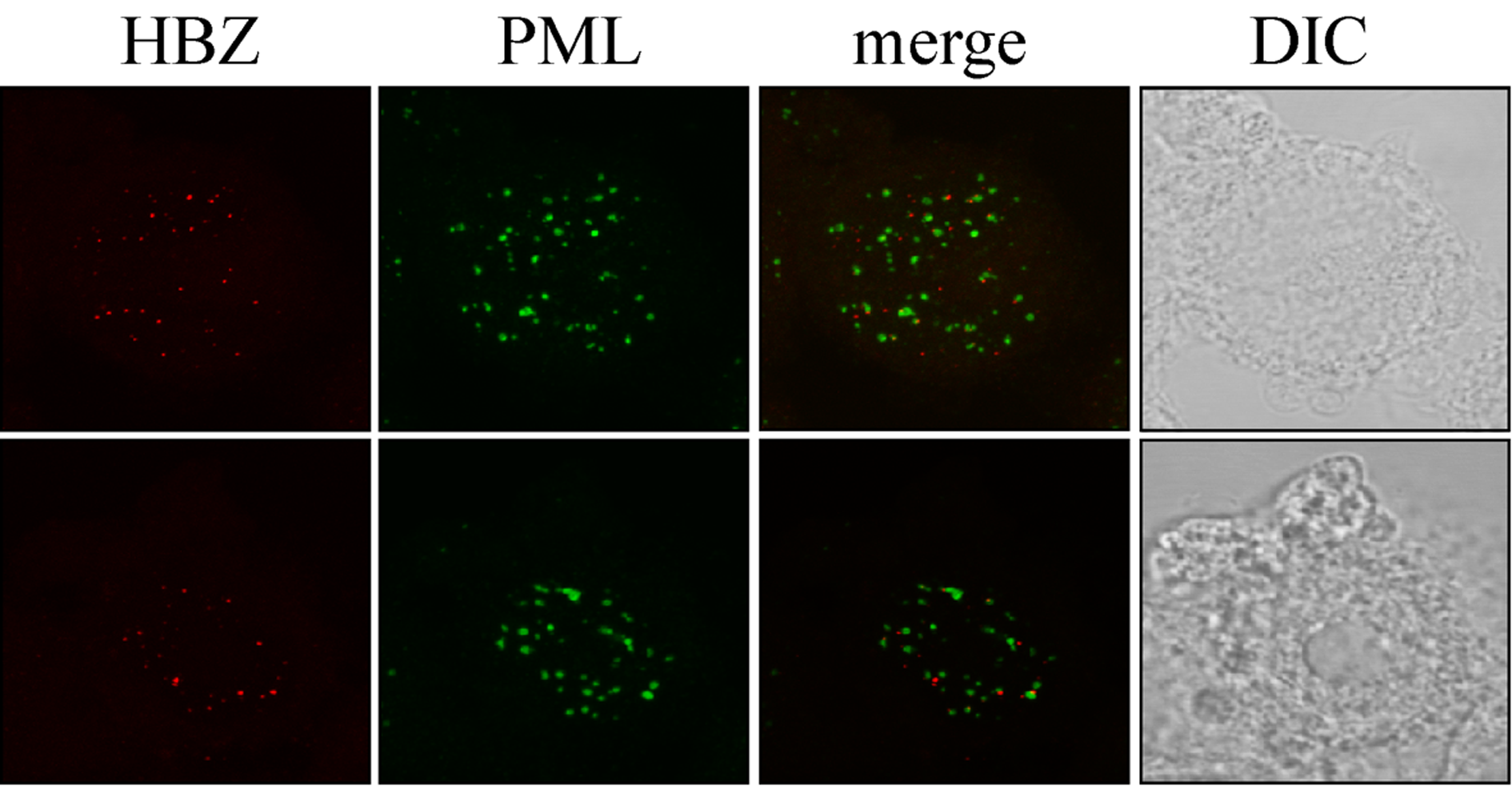

Supplement: Additional file 3 — Figure S3. HBZ nuclear speckles do not co-localize with PML nuclear bodies (PML-NB). ATL-2s cells were reacted in a pairwise combination with the 4D4-F3 ant-HBZ mAb antibody and a polyclonal rabbit anti-PML antiserum. Anti-HBZ mAb was revealed by Alexa fluor 546 labeled goat anti-mouse IgG (red), whereas the rabbit antibodies were revealed by Alexa fluor 488-labeled goat anti-rabbit antibodies (green). Two representative cells are shown. The “merge” column panels represent the merge between the Alexa fluor 546 and the Alexa fluor 488 signals. A co-localization of HBZ with PML would result in a yellow color. Differential interference contrast (DIC) image of each cell analyzed is shown in the last column panels. [file 12977_2015_186_MOESM3_ESM.tif]
